# Process and Results of Implementing Disease Management Program in Patients with First-time Ischemic Stroke

**Published:** 2018-07

**Authors:** Hong-Rong YANG, Lei MA, Yi-Feng JIANG, Yun-Cheng WU, Eugene C. LAI, Yan-Hong ZHU

**Affiliations:** 1. Dept. of Science, Shanghai General Hospital, Shanghai Jiao Tong University School of Medicine, Shanghai, China; 2. Dept. of Neurology, Shanghai General Hospital, Shanghai Jiao Tong University School of Medicine, Shanghai, China; 3. Houston Methodist Neurological Institute, Houston, Texas, USA; 4. Health Economic Research Institute, Shanghai Jiao Tong University National Institute Hospital Development, Shanghai, China; 5. School of Public Health, Shanghai Jiao Tong University School of Medicine, Shanghai, China

**Keywords:** Disease management program, Ischemic stroke, Average length of stay, Cost-effectiveness analysis

## Abstract

**Background:**

This study aimed to examine the effect of disease management program (DMP) on the patients with first-time ischemic stroke (IS).

**Methods:**

A DMP with 4 parts of performance indicators (PIs, including outpatient, emergency department, inpatient and follow-up treatment) was implemented in patients with stroke in 2 hospitals (Hospital T and R) in Shanghai China from 2007–2010. The effect of DMP on the outcome of IS patients was analyzed according to the criteria of the National Institute of Health Stroke Scale (NIHSS). Furthermore, the total effective rate of DMP, average length of stay, hospitalization cost, and cost-effectiveness ratio (CER) between DMP and non-DMP patients were calculated, followed by the cost-effectiveness analysis.

**Results:**

The total effective rate of DMP (T: 69.9%; R: 76.6%) was significantly (*P*<0.05) higher than that of non-DMP (T: 60.8%; R: 62.7%) group in the same hospital. In addition, a significant (*P*<0.05) difference in effective rate was observed between DMP and non-DMP at the NIHSS score ≥ 7. Furthermore, the average length of stay and hospitalization cost of the patients in DMP group were significantly (*P*<0.05) lower than those in non-DMP group. A superior CER was also found in DMP group than non-DMP group.

**Conclusion:**

The implementation of DMP for IS can effectively improve the treatment outcome and reduce the average length of stay and hospitalization cost.

## Introduction

Stroke is a global health problem and the main cause of disability-adjusted life-years according to the previous report (1). Multiple factors, including sociodemographic, clinical, psychosocial, and behavioral factors can impact the onset and progression of stroke (2). Among them, behavioral risk factors are responsible for 80% of all the stroke cases (3). Therefore, integrated health care approaches which can better manage the high-risk behaviors of stroke patients are urgently needed.

Several approaches have been proposed to reduce rehospitalization and healthcare costs (4). Disease management program (DMP) appears to be a multifaceted program associated with improving health outcomes and lowering costs among chronic diseases (5). This program is characterized by disease staging, evidence-based guidelines, patient education, collaborative care, aggressive screening for complications, and early and appropriate specialty referral (6). In recent years, a number of DMPs have been examined, and favorable effects on morbidity and even mortality have been reported (7, 8). For instance, the association of evidence-based guidelines with stroke care was examined and suggested a statistically and clinically significant improvement in the treatment of patients with IS (9). Although certain progress has been made for the use of DMP, further verification of the short-term and long-term effects on patient outcomes is still needed.

Therefore, the present study was designed to assess the short-term effects of DMP implementation on the outcomes of patients with IS. Briefly, the DMP was implemented in patients with stroke in two hospitals in Shanghai. The effect of DMP on the outcome of IS patients was analyzed according to the criteria of the National Institute of Health Stroke Scale (NIHSS). Additionally, the average length of stay, hospitalization cost, and cost-effectiveness of IS patients under the implementation of DMP were analyzed.

## Materials and Methods

### Study Population

This is a prospective and observational study. During Aug 1, 2007, to Jul 31, 2010, the IS patients in Hospital T (a teaching hospital) and Hospital R (a regional hospital) were recruited in our study according to the following inclusion criteria: (1) ≥ 18 yr old; (2) received a final diagnosis of IS within two weeks after their stroke.

Patients were excluded if they (1) had no neurological functional deficit (i.e. National Institutes of Health Stroke Scale (NIHSS) score = 0); (2) were transferred from another hospital; (3) were refractory to repeated outpatient treatments; (4) were re-hospitalized within 30 d from a previous discharge, or had neurological deficits from a previous stroke.

The study was approved by Shanghai General Hospital Ethics Committee and performed in accordance with the ethical standards.

### Implementation of DMP

There were four parts of performance indicators (PIs) in the DMP issued by Chinese Hospital Association, including outpatient, emergency department, inpatient, and follow-up treatments (Table 1). In this study, seven core measurements in DMP were designated as key PIs for IS patients according to evidence-based medicine and actual treatment process, including computed tomography (CT)/magnetic resonance imaging (MRI) scan within 45 min after admission, antiplatelet therapy prescribed within 48 h after hospitalization, anticoagulation therapy for patients with atrial fibrillation, lipid assessment, statin therapy for patients with dyslipidemia, dysphagia assessment, and vascular function assessment within 24 h after hospitalization. The monitoring and evaluation of PIs were achieved by their physicians. The patients who accomplished all the seven measurements were assigned to DMP group, while the patients failing to receive one or more of the core measurements were classified to non-DMP group.

**Table 1:** Performance indicators (PIs) for ischemic stroke (IS) during disease management program (DMP)

**Diagnosis and treatment process**	**Performance indicator**	**Definition of indicator accomplishment**
Outpatient and emergency treatment	CT/MRI scan within 45 min after admission^*^	/
	Thrombolysis therapy	Thrombolytic therapy using tissue plasminogen activator (rt-PA) or urokinase within 6 h after symptom onset for patients with appropriate indications
Inpatient treatment	Antiplatelet therapy prescribed within 48 h after hospitalization^*^	/
	Anticoagulation therapy for patients with atrial fibrillation ^*^	/
	Lipid assessment^*^	/
	Statin therapy for patients with dyslipidemia^*^	Statin therapy for patients with abnormal blood lipid (LDL >2.6 mol/L)
	Dysphagia assessment ^*^	/
	Health education	/
	Vascular function assessment within 24 h after hospitalization^*^	Vascular function assessment within 24 h after hospitalization, including carotid ultrasound, TCD, CTA or MRA
		/
Post-discharge follow-up	Antiplatelet therapy prescribed upon discharge	Post-discharge administration of aspirin or clopidogrel for secondary stroke prevention

* Key PIs for IS during DMP.

CT: computed tomography; MRI: magnetic resonance imaging; LDL: Low-density lipoprotein; TCD: transcranial ultrasound; CTA: computed tomography angiography; MRA: magnetic resonance angiography

### Outcome, Average Length of Stay, Cost and Cost-effective Assessment

Outcome evaluation was carried out to confirm the effectiveness of the therapy when patients were discharged from the hospital. The severity of IS at the time of admission and discharge was assessed according to the criteria of NIHSS by two experienced neurologists who received the same training for the evaluation. The NIHSS is generally reliable and is accepted widely for measuring acute stroke deficits in clinic (10). The score of neurological functional deficits was determined according to the following criteria (11): NIHSS score = 1 was defined as mild; 2 ≤ NIHSS score < 6 was defined as moderate; NIHSS score ≥ 7 was defined as severe. The outcome assessment criteria were according to the reduction in cumulative score of NIHSS: effective management was defined as NIHSS score decreasing > 17%, and ineffective management was defined as NIHSS score decreasing ≤ 17%. The effective rate of DMP referred to total effective number corresponding to total number of patients (total effective number/total number of patients), taken as the effectiveness indicator.

In addition, the average length of stay was calculated. The hospitalization cost was taken as the cost indicator.

A cost-effective analysis for DMP implementation was carried out by using the Bootstrap method according to the previous description (12, 13). The ratio of cost to one percent increments of the effective rate (cost/one percent increments of the effective rate) was calculated as cost-effectiveness ratio (CER). Additionally, Bootstrap method (14) was also used to analyze the distribution of incremental cost-effectiveness ratios (ICERs) (ΔC/ΔE).

### Statistical Analysis

All the statistical analyses were performed using the SPSS 17.0 software (Chicago, IL, US). The t-test or rank-sum test was used to compare mean (normally distributed) or median (not normally distributed) between different groups respectively. The χ^2^ test and Mantel-Haenszel test were used to analyze continuous variables and categorical variables. Bootstrap method was used to perform cost-effectiveness and incremental cost-effectiveness analyses. For all analyses, a two-tailed *P* < 0.05 was considered as significant.

## Results

### Baseline Data in DMP Group and non-DMP Group

Total 249 and 469 cases in Hospital T were respectively assigned to DMP Group and non-DMP Group, while in Hospital R, 128 and 343 cases were assigned to DMP Group and non-DMP Group respectively. The response rate in this study was 100%. In addition, no statistical difference was found on demographic characteristics, medical-expense payment, co-morbid disease or severity at time of initial onset, while statistical difference was found on time of disease onset between DMP group and non-DMP group in Hospital T and Hospital R (Table 2).

**Table 2:** Baseline data in DMP group and non-DMP group

		**Hospital T**			**Hospital R**		
		DMP group (n_Ad_ = 249)	non-DMP group (n_Au_ = 469)	*P*	DMP group (n_Bd_ = 128)	non-DMP group (n_Bu_ = 343)	*P*
Demographic characteristics							
Man (%)		160 (64.3)	271 (57.8)	0.09	77 (60.2)	178 (51.9)	0.11
Age (%)							
	18–45 yr	6 (2.4)	4 (0.9)	0.13	1 (0.8)	8 (2.3)	0.58
	45–64 yr	97 (39.0)	167 (35.6)		39 (30.5)	96 (28.0)	
	≥ 65 yr	146 (58.6)	298 (63.5)		99 (68.3)	239 (69.7)	
Smoke (%)		140 (57.4)	278 (64.1)	0.09	81 (63.4)	218 (63.7)	0.91
Drink (%)		176 (73.0)	336 (78.3)	0.12	91 (72.2)	253 (74.0)	0.70
Disease time (%)							
One year before implementation		23 (9.2)	220 (46.9)	< 0.01^*^	13(10.2)	115 (33.5)	< 0.01^*^
One year after implementation		89 (35.7)	146 (31.1)		56 (43.8)	89 (25.9)	
Two years after implementation		137 (55.0)	103 (22.0)		59 (461)	139 (40.5)	
Medical-expense payment (%)							
Medical Insurance		205 (82.7)	387(82.5)	0.96	121 (94.5)	322 (93.9)	0.79
Self-paying		43 (17.3)	82 (17.5)		7 (5.5)	21 (6.1)	
Co-morbid disease (%)							
Hypertension		186 (74.7)	337 (71.9)	0.42	91 (71.1)	239 (69.7)	0.77
Hyperlipidemia		42 (16.9)	78 (16.6)	0.94	4 (3.1)	14 (4.1)	0.79
Diabetes		66 (26.5)	138(29.4)	0.41	40 (31.1)	80(23.4)	0.08
Severity at time of initial onset (NIHSS score)—M (Q1-Q3)^※^		4 (2–6)	3 (2–6)	0.99	2 (1–4)	3 (1–7)	0.20

※ Rank sum test (S) between severity at time of initial onset at Hospital T and Hospital R (because the original data were measurement data which did not conform to the normal distribution) DMP: Disease Management Program

* *P* < 0.05

### Implementation of PIs in DMP for IS

As shown in Table 3, accomplishment rates of the PIs in DMP for IS patients were significantly increased two years after the DMP implementation in both hospitals (*P*<0.01). Of all PIs, the accomplishment of thrombolysis in the patients from Hospital T was increased after one year implementation while thrombolysis was decreased in Hospital R in the second year (*P*<0.05). For the accomplishment rates of CT/MRI scan within 45 min after admission and lipid assessment, there was no significant difference among three groups in both hospitals. Furthermore, the accomplishment rates of other PIs increased year by year in Hospital T while those were increased at the first year and stayed at the same rate in the second year in Hospital R.

**Table 3:** Accomplishing rate (%) of the performance indicators before and after implementation of disease management program (DMP) for ischemic stroke (IS)

**Group**	**Hospital T**				**Hospital R**			
	One year before implementation	One year after implementation	Two years after implementation	*P*	One year before implementation	One year after implementation	Two years after implementation	*P*
CT/MRI scan within 45 min after admission^▵^	97.1	98.7	97.9	0.47	98.4	97.9	98	0.94
Thrombolytic therapy	0	1.3	3.8	< 0.01^*†^	15.6	10.3	6.6	0.03^*†^
Anticoagulation therapy for patients with atrial fibrillation^▵⋆^	41.2	70.6	59.4	0.04^*‡^	53.8	85.2	79.1	< 0.01^*§^
Antiplatelet therapy within 48 h after hospitalization^▵^	57.2	89.8	93.8	< 0.01^*§^	91.4	90.3	90.9	0.96
Lipid assessment^▵^	98.8	97.0	98.3	0.37	96.9	96.5	97	0.97
Statin therapy for patients with dyslipidemia^▵⋆^	24.9	54.8	58.5	< 0.01^*§^	79.2	76.9	62.9	0.07
Dysphagia assessment^▵^	86.8	95.3	95.8	< 0.01^*§^	65.6	99.3	99	< 0.01^*§^
Vascular function assessment within 24 h after hospitalization^▵^	35.4	66.0	77.9	< 0.01^*¶^	21.9	48.3	44.9	< 0.01^*§^
Health education	0.4	13.2	93.3	< 0.01^*¶^	44.5	97.2	100	< 0.01^*§^
Antiplatelet therapy upon discharge	71.2	86.0	92.9	< 0.01^*§^	93	91	92.4	0.73
All key indicators (above-mentioned indicator marked with^▵^)	9.5	37.9	57.1	< 0.01^*¶^	10.2	38.6	29.8	< 0.01^*§^

*† represent accomplishing rate comparison among three groups. Significant difference was found between one year before implementation and two years after implementation.

*‡ represent accomplishing rate comparison among three groups. Significant difference was found between one year before implementation and one year after implementation.

*§ represent accomplishing rate comparison among three groups. Significant difference was found between one year before implementation and one year after implementation as well as between one and two years after implementation.

*¶ represent accomplishing rate comparison among three groups. Significant difference was found between any two groups.

▵⋆ for anticoagulation therapy indicator, the denominator is patients with atrial fibrillation; for statin therapy indicator, the denominator is patients with dyslipidemia.

▵ = key performance indicator.

CT: computed tomography; MRI: magnetic resonance imaging

### Comparison of Outcome Improvement between DMP Group and non-DMP Group

The total effective rates of DMP and non-DMP were 69.9% and 60.8%, respectively in Hospital T. In Hospital R, the total effective rates were 76.6% and 62.7% for DMP and non-DMP group. Significant differences were found between DMP and non-DMP group in both hospitals (*P*_*T*_=0.018, P_R_=0.004).

The NIHSS scores at initial onset of IS were used as the stratification factor to compare the outcome between DMP and non-DMP group in two hospitals (Table 4). Estimated values of inter-group combined relative risk (RR) between DMP and non-DMP in Hospital T and Hospital R was 1.27 and 1.38, respectively. Significant difference on RR was found between DMP and non-DMP. For the patients with NIHSS score ≥ 7 (most severe), significant outcome improvement was observed in DMP group than that in non-DMP group (*P*_*T*_=0.01, P_R_<0.01).

**Table 4:** Outcome comparison between disease management program (DMP) group and non-DMP group in the hospital using initial onset symptom severity as stratification factor

**Initial onset severity (NIHSS score)**	**Group**					**Hospital R**			
			Ineffective	RR_i_ (95% CI)	*P*	Effective	Ineffective	RR_i_ (95% CI)	*P*
Mild (score 1)	DMP Group_(1)_	14	14	1.55 (0.924–2.599)	0.11	12	14	1.096 (0.670–1.793)	0.72
	non-DMP Group_(1)_	20	42			32	44		
Moderate (score 2–6)	DMP Group_(2–6)_	117	49	1.05 (0.923–1.187)	0.48	62	16	1.168 (1.002–1.361)	0.06
	non-DMP Group_(2–6)_	202	98			115	54		
Severe (score ≥7)	DMP Group_(≥7)_	43	12	1.33^*^ (1.075–1.640)	0.01	24	0	1.441^*^ (1.264–1.644)	< 0.01
	non-DMP Group_(≥7)_	63	44			68	30		

* Significant difference was found between DMP and non-DMP group; RR: risk ratio.

Effective management = Decrease NIHSS score by 17% to 100%.

Ineffective management = Decrease NIHSS score at least than 17% or worse

### Comparison of Average Length of Stay between DMP Group and non-DMP Group

A trend test was performed between the average length of stay and DMP implementation in Hospital T and R and a linear correlation was found. Decrease in the average length of stay was found in Hospital T and R with the progression of the DMP implementation. A significant difference was found in the average length of stay between DMP (16.5) and non-DMP (21.5) group (*P*=0.007) before one year implementation of DMP in Hospital T. However, no significant difference was found in the average length of stay between DMP and non-DMP group after one year implementation of DMP in Hospital T and hospital R (Table 5).

**Table 5:** Average length of stay with different implementation progress in Hospital T and Hospital R

	**Hospital T**				**Hospital R**			
	Average length of stay^*^ (Days)	DMP Group (Days)	non-DMP Group (Days)	*P*	Average length of stay^*^ (Days)	DMP Group (Days)	non-DMP Group(Days)	*P*
One year before implementation (n_1_ = 371)	21	16.5	21.5	0.007	21.9	19.2	22.5	0.261
One-year implementation (n_2_ = 380)	16.4	17	16.7	0.404	20	19.2	20.9	0.296
Two-year implementation (n_3_ = 438)	15.6	16.6	16.3	0.168	17.9	17.8	18.2	0.716

* Trend test between the average length of stay at Hospital T and Hospital R and implementation progress (*P* < 0.0001); DMP: disease management program.

### Comparison of Hospitalization Cost between DMP Group and non-DMP Group

The costs of the medical procedures (except for B mode ultrasound) and treatment, and the costs of hospital stay remained essentially unchanged during the study period. The cost incurred was not corrected because the changes in consumer prices index (CPI) were negligible. The hospitalization cost in DMP group was significantly lower than that in non-DMP group in Hospital T (*P*=0.013) while no significant difference was found in DMP and non-DMP group in Hospital R (Table 6).

**Table 6:** Hospitalization cost before and after implementation of disease management program (DMP) for ischemic stroke (IS) (in CNY)

**Cost item**	**Hospital T**			**Hospital R**		
	DMP Group	non-DMP Group	t value	DMP Group	non-DMP Group	t value
Wards	612	701	5.47^*^	613	778	3.22^*^
General treatment	830	687	2.38^*^	883	1041	1.25
Care	180	201	4.27^*^	228	242	0.85
Assay	1426	1253	3.48^*^	2509	2394	0.67
Medication	5850	7779	5.76^*^	5643	5889	0.12
Oxygen	45	126	4.64^*^	315	337	0.24
Examination	1261	862	8.76^*^	2187	1915	3.57^*^
Other	1113	931	2.87^*^	537	565	0.22
Total costs	12499	13581	2.50^*^	12640	12942	0.45

* *P* < 0.05; CNY: Chinese Yuan

### Cost-effectiveness Analysis between DMP Group and non-DMP Group

In Hospital T, the CER in DMP and non-DMP group were respectively 178.8 and 223.4, and a significant difference was found between these two groups (*P*<0.05). In Hospital R, there was also a significant difference in CER between DMP (165.0) and non-DMP (206.4) group (*P*<0.05).

**Fig. 1:**
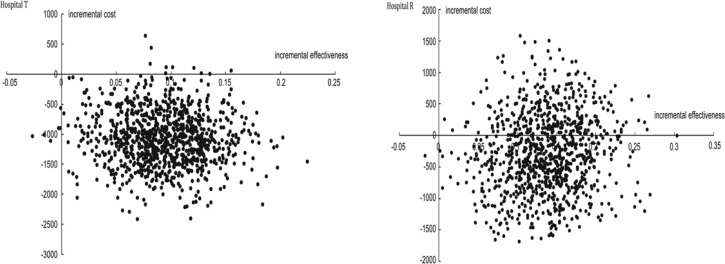
Bootstrap figure of incremental cost-effectiveness ratio (ICER) for disease management program (DMP) group and non-DMP group in Hospital T and Hospital R

In addition, a bootstrap map of ICERs for the DMP implementation in IS patients in both hospitals was constructed. ICERs was −118.9 for Hospital T, and 98.5% of scattered dots of ICERs were located in the fourth quadrant (Fig. 1). Compared with non-DMP Group, a decreasing on the hospitalization cost and an increasing on effective rate were found in DMP Group of hospital T. In Hospital R, ICERs was −21.7, and 31.5% of the scattered dots of ICERs were located in the first quadrant while 68.4% in the fourth quadrant.

## Discussion

DMP has been implemented in several countries and diseases (7, 15–17), and achieved the optimal disease management. Since 2007, Chinese Ministry of Health has implemented the DMP for IS patients in teaching and regional hospitals (18). In the present study, the implementation of DMP significantly improved the prognosis and outcome of IS patients, as well as decreased the total cost.

After DMP implementation for IS in two hospitals, the accomplishment rates of the key IPs were improved. However, the improvement was not consistent between two hospitals. The difference may be attributed to the inappropriate usage of thrombolytic drugs beyond the qualifying indications. After implementation of DMP for IS patients, the thrombolytic indications were more strictly followed, and the accomplishment rate of thrombolysis was decreased. Therefore, the implementation of DMP may make the diagnosis and treatment more scientific and consistent. The measures to expedite clot lysis and restore circulation may decline the extent of brain injury and improve outcome after stroke (19). According to the requirement of DMP, thrombolysis should be performed within 4.5 h after the onset of stroke (20). Therefore, the improvement of the accomplishment rates of IPs including thrombolysis may finally improve the outcome of IS. Dartmouth-Hitchcock Medical Center has implemented standardized treatment of IS since 2004, and the average accomplishment rate of the seven key PIs in 2009 was 92.6% (21) which was higher than that in two hospitals of our study. Therefore, more efforts are still needed to further develop DMP for IS in China.

In general, early signs and symptoms of disease are not recognized by patients. However, symptomatic deterioration continues will finally lead to unnecessary hospitalizations. DMP can prevent exacerbation of a disease, resulting in the decrease of the average length of stay (22). Moreover, the implementation of DMP also ensured the strict completion of the requirements in the average length of stay, which caused the decline of the average length of stay for IS patients in both hospitals. Additionally, the patient education program, belonging to DMP, is successful in improving health status, and decreasing rates of hospitalization (23). The patient education program can provide patients with necessary knowledge and confidence (self-efficacy) to deal with disease-related problems (24). Systematic screening, another aspect of the DMP, has also been found to increase the proportion of patients receiving appropriate treatment compared to usual care (25).

The goal of DMP is to improve health outcomes and simultaneously reduce healthcare cost (26). In our study, hospitalization cost in DMP group was significantly lower than that in non-DMP group, which may be partly due to the optimization of the average length of stay (22). Moreover, ICER analysis indicated that both hospitalization cost and therapeutic benefits were better controlled in DMP group than that in non-DMP group in Hospital T. In Hospital R, the therapeutic benefits were better controlled while the hospitalization cost for most patients was reduced, which suggested that the implementation of DMP extended an economic efficiency because the improvement on therapeutic effectiveness did not increase the hospitalization cost for most of patients.

However, there are several potential limitations in our study. On one hand, some medical records data on outpatient and emergency treatment costs were not detailed during hospitalization. On the other hand, the long-term therapeutic outcome of DMP implementation for IS was not studied in the present study, but it will be investigated in the near future.

## Conclusion

The implementation of DMP for IS can obviously improve the standard use of thrombolytic drugs, effectively improve the treatment outcome, and decrease the average length of stay and the hospitalization cost. Taken together, DMP will have a wide application prospect and will give some hints to policymakers.

## Ethical considerations

Ethical issues (Including plagiarism, informed consent, misconduct, data fabrication and/or falsification, double publication and/or submission, redundancy, etc.) have been completely observed by the authors.
